# Endoscopic Incision Therapy for Benign Pyloric Stricture

**DOI:** 10.14309/crj.0000000000000855

**Published:** 2022-08-31

**Authors:** Jeongmin Choi

**Affiliations:** 1 Department of Internal Medicine, Division of Gastroenterology, Sanggye Paik Hospital, Inje University College of Medicine, Nowon-gu, Seoul, Korea

## Abstract

We report that an 82-year-old woman with benign pyloric stricture and fibrous scars around the pylorus was safely treated with endoscopic incisional therapy using an insulation-tipped knife. At the 1-year follow-up endoscopy, the pylorus was open.

## CASE REPORT

Peptic ulcer disease is the leading cause of benign gastric outlet obstruction (GOO). Caustic ingestion, inflammatory diseases, and nonsteroidal anti-inflammatory drugs can also cause benign GOO.

Endoscopic balloon dilation is the first-line therapy for benign pyloric stricture, but multiple dilation procedures are sometimes required to achieve optimal lumen dilatation, and there is a potential risk of perforation.^1,2^ Alternatively, endoscopic incision therapy is used to treat anastomotic strictures after esophagectomy.^3,4^

**Figure 1.**
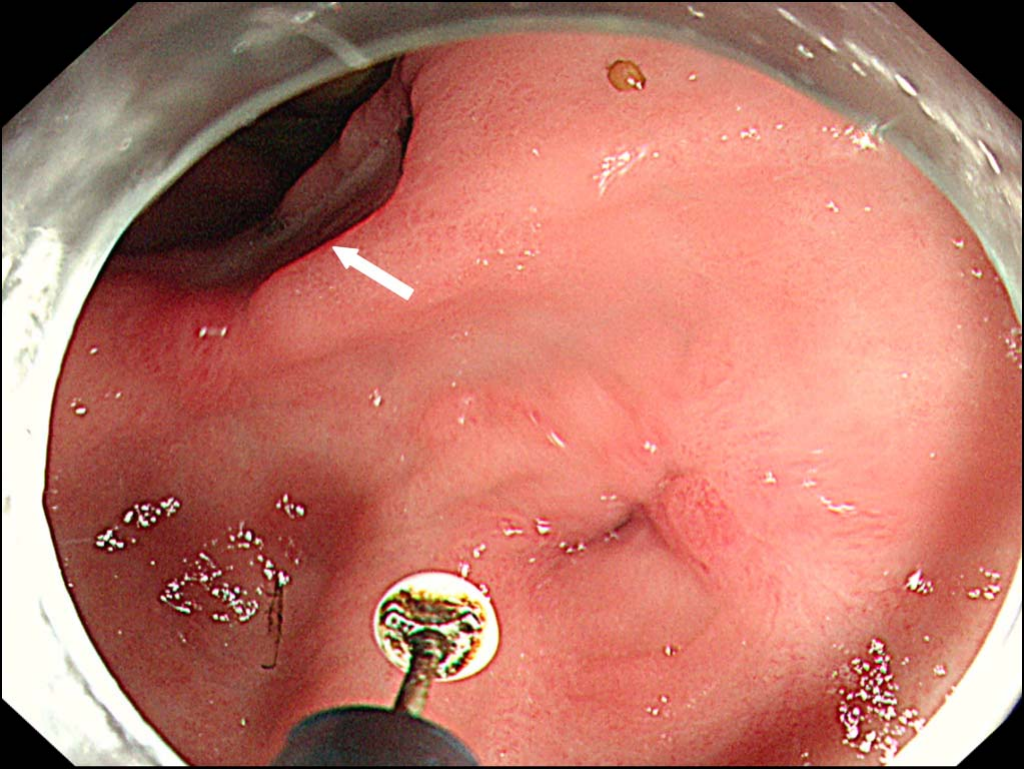
Endoscopic incision therapy for benign pyloric stricture. The near-closed pyloric stricture with surrounding scars and insulation-tipped knife were observed. An active ulcer was also observed at the gastric angle (arrow).

In this article, we present a case of endoscopic incision therapy for benign pyloric stricture. An 82-year-old woman presented with hematemesis and dizziness. She was bedridden after a stroke and weighed 41 kg. Esophagogastroduodenoscopy revealed a benign pyloric stricture with ulcer scars around the pylorus (Figure 1). Endoscopic biopsy revealed benign gastritis, and *Helicobacter pylori* was not detected.

**Figure 2.**
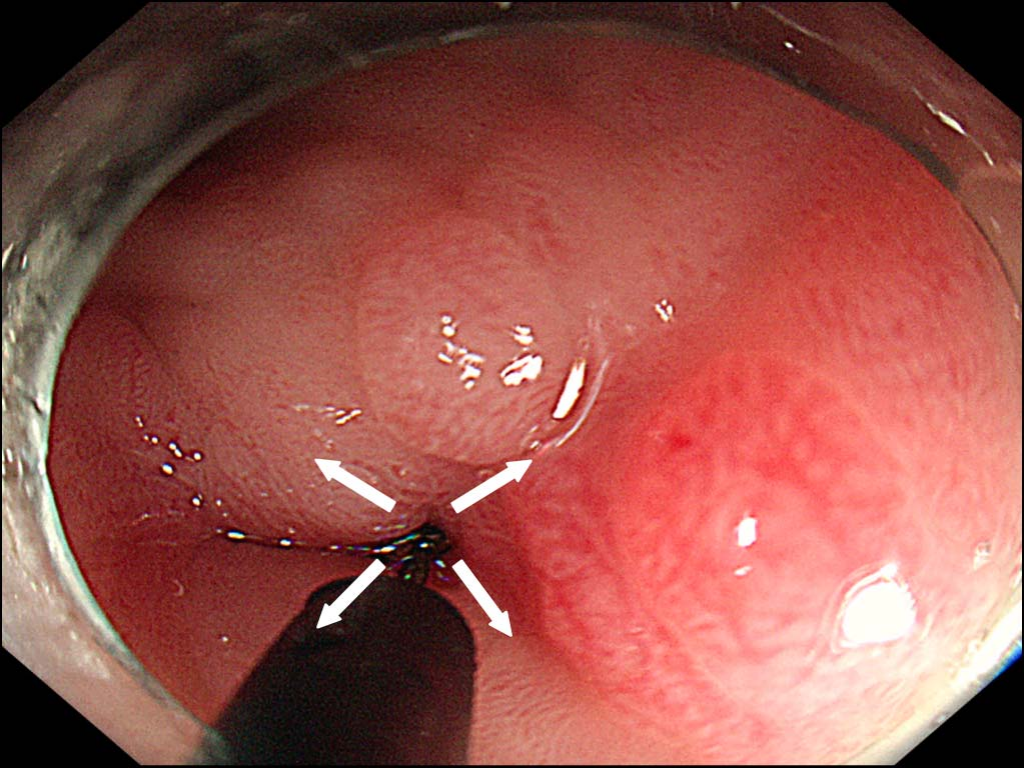
Endoscopic incisions were made in the quadrant of the pyloric channel.

**Figure 3.**
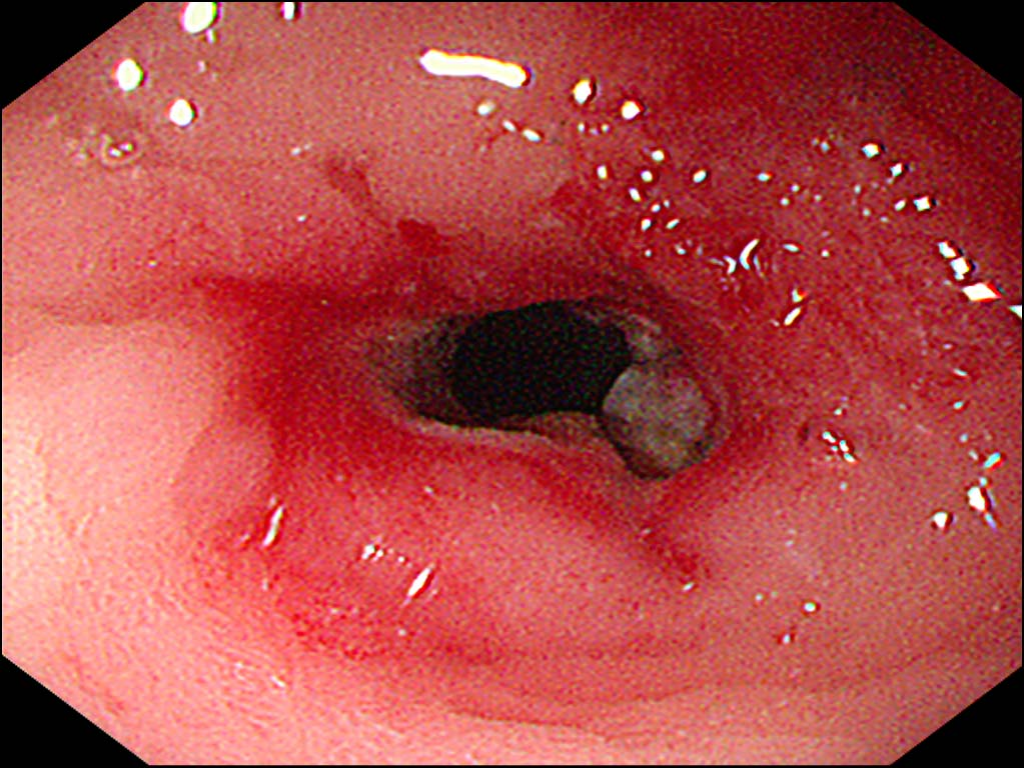
The endoscope could not pass through the stricture after incision in the pyloric channel.

**Figure 4.**
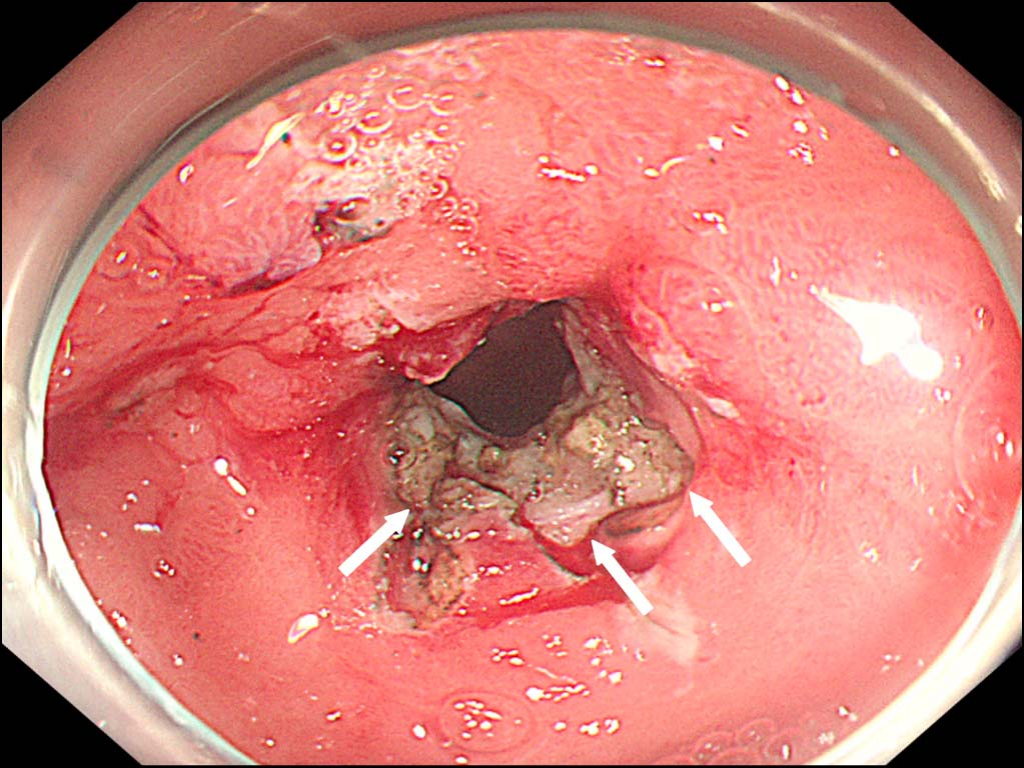
After incision in the fibrous scar around the pylorus (arrows), the endoscope was able to pass through the stricture.

Because she was bedridden and cachexic, safety was a top priority when choosing a treatment option. Owing to the risk of perforation during endoscopic balloon dilation therapy, we decided to perform endoscopic incision therapy as an alternative. Insulation-tipped knife 2 (Olympus Korea) was used for incision. The electrosurgical unit was VIO 300D (ERBE, Germany), and the ENDO CUT I mode (effect 2, duration 2, interval 3) was used. Endoscopic incisions were made in the quadrant of the pyloric channel (Figure 2). After the incision, the scope could not pass through the stricture, which indicated additional incisions to be made in the fibrous scar tissue around the pylorus (Figure 3). Then the scope was able to pass through the stricture (Figure 4; see Video, Supplementary Digital Content, http://links.lww.com/ACGCR/A27).

**Figure 5.**
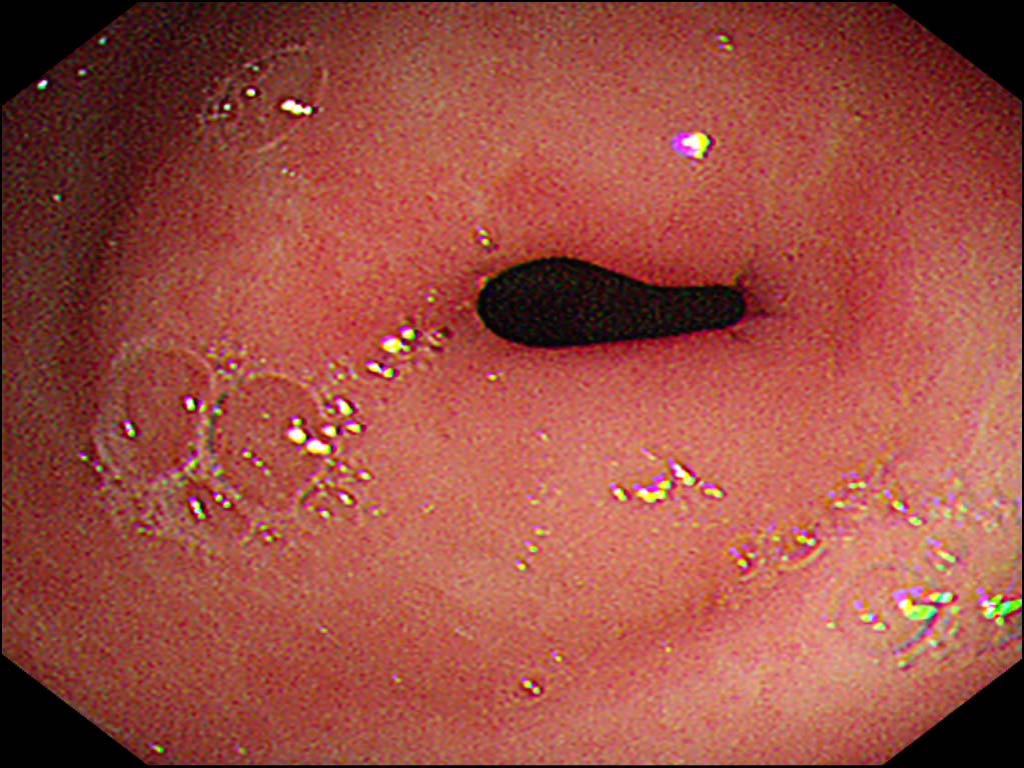
One-year follow-up upper endoscopy. The pyloric channel is open and dilated.

**Figure 6.**
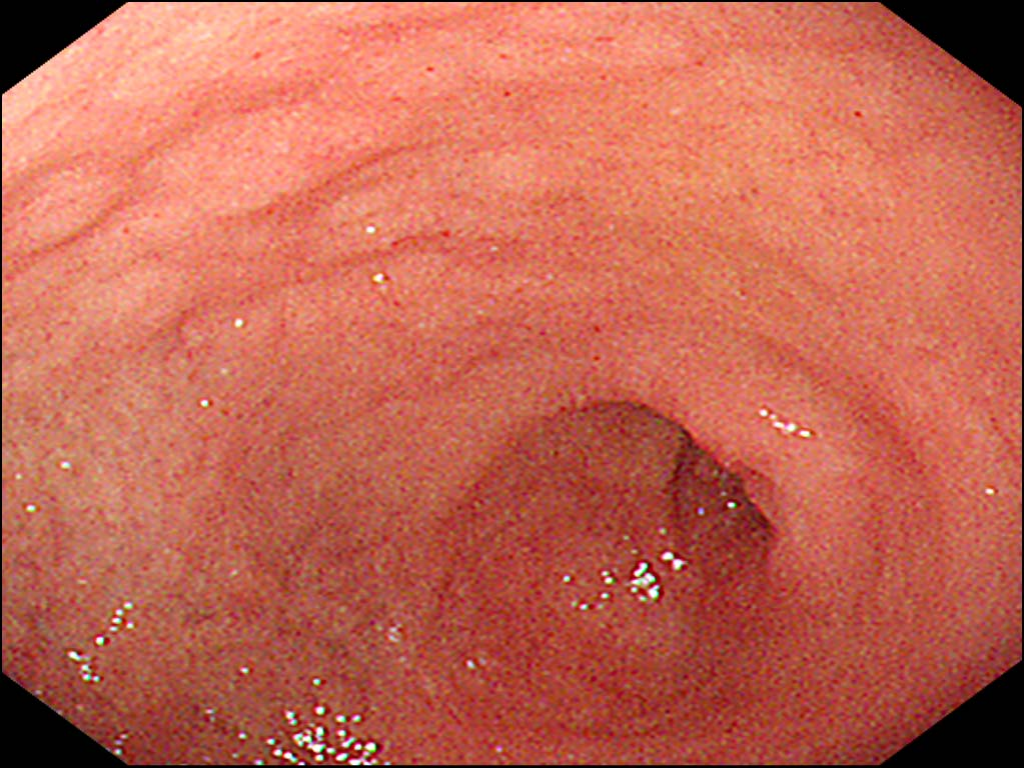
The endoscope is able to reach the duodenal lumen without any resistance.

She was able to eat a solid diet and gained 8 kg in 3 months. At the 1-year follow-up esophagogastroduodenoscopy, the pylorus was open and dilated, and the endoscope reached the duodenum without any resistance (Figures 5 and 6).

## DISCUSSION

Treatment may vary depending on the etiology of benign GOO and available expertise. In addition to endoscopic balloon dilatation and incision, intralesional steroids combined with balloons can be used to inhibit stricture formation.^5^

Endoscopic balloon dilation for pyloric stricture shows an overall technical success rate of 85% and a long-term patency rate of 70%.^1^ However, many patients require multiple cessions of balloon dilation, and half of the patients ultimately require surgery after dilation.^2^ Regarding safety, Kozarek et al reported 1 perforation in 23 patients and Lau et al reported 4 perforations in 54 patients.^1,2^ A large 20 mm balloon dilation resulted in 2 perforations in 3 patients.^2^ However, even a small 10 mm balloon can lead to perforation, necessitating surgery.^6^

Endoscopic incision therapy is a straightforward procedure under a direct endoscopic view. It can selectively cut the fibrous tissue, avoiding the normal muscle layer. The extent of the incision depends on the severity of the stricture. The endoscopist cut through the stricture of the pyloric channel, but the endoscope could not pass through the stricture because of the fibrous scar around the pylorus. Next, the incision was extended to the fibrous scar around the pylorus to allow the endoscope to pass through.

Few studies have been reported on endoscopic incision therapy for refractory pyloric stenosis.^7,8^ Three patients with pyloric stenosis who failed treatment with balloon dilatation were successfully treated using a sphinctertome.^7^ Five patients with postoperative pyloric stenosis were treated with a combination of balloon dilatation and needle-type knife incisions.^8^ Short-term efficacy of incisional therapy is good, but long-term lumen patency should be validated.

## DISCLOSURES

Author contributions: J. Choi is a sole author and is the article guarantor.

Financial disclosure: None to report.

Informed consent was obtained for this case report.
